# Therapeutic implications of structural and functional neuroimaging findings in delusional disorder: A case report and review of literature

**DOI:** 10.1192/j.eurpsy.2021.1382

**Published:** 2021-08-13

**Authors:** G.F. Fucho, A. González-Rodríguez, A. Alvarez Pedrero, A. Guàrdia, L. Delgado, J. Cobo, S. Acebillo, J.A. Monreal, D. Palao Vidal, J. Labad

**Affiliations:** 1 Mental Health, Parc Taulí University Hospital. Autonomous University of Barcelona (UAB). I3PT, Sabadell, Spain; 2 Mental Health, Parc Taulí University Hospital. Autonomous University of Barcelona (UAB). I3PT, CIBERSAM, Sabadell, Spain; 3 Mental Health, Hospital of Mataró. Consorci Sanitari del Maresme. CIBERSAM., Mataró, Spain

**Keywords:** Brain imaging, Delusional disorder, Paranoia, Brain changes

## Abstract

**Introduction:**

Several neuroimaging studies on psychosis spectrum have been published in the last decades, most of them based on schizophrenia. In the context of neuroanatomical dysfunctions, clinical and prognosis implications have been reported. Nevertheless, only a few studies have been focused on delusional disorder (DD).

**Objectives:**

To present the case of a patient diagnosed with DD who suffered from two cerebrovascular events after the onset of the psychiatric disease. Our aim is to elucidate potential implications of those lesions on the course of DD. We also reviewed the literature to assess evidence for specific changes in DD on brain structures and functions.

**Methods:**

Case report and non-systematic narrative review in PubMed (2000-2020).

**Results:**

Case report: A 66-year-old female with DD presenting, during the course of the disease, general atrophy and consecutive ischemic lesions on parietal, occipital and cerebellar areas. Clinical stabilization was achieved 12-16 months after risperidone 1.5mg/day treatment. Review: 19 studies were included: Structural brain data (n=15), Functional data (n=13). Most of the structural neuroimaging studies reported white and gray matter abnormalities, particularly in temporal, parietal and frontal lobes, and in limbic structures. Functional neuroimaging studies pointed to temporal and parietal lobes, as well as basal ganglia and limbic related structures.

**Conclusions:**

Temporal, parietal, frontal, basal ganglia and limbic-related structures, as well as dysfunctions in other specific brain regions, may be implicated in the core symptoms of DD. These findings might be further investigated as potential neuroimaging markers of prognosis, such as partial or delayed response to antipsychotic treatment, as presented in our case.

